# Evaluation of Systemic Renin and Angiotensin II Levels in Normal Tension Glaucoma

**DOI:** 10.3390/jcm9123838

**Published:** 2020-11-26

**Authors:** Soo Ji Jeon, Hyung Bin Hwang, Na Young Lee

**Affiliations:** 1 Department of Ophthalmology, Eunpyeong St. Mary’s Hospital, College of Medicine, The Catholic University of Korea, Seoul 03312, Korea; sj8801@gmail.com; 2 Department of Ophthalmology, Incheon St. Mary’s Hospital, College of Medicine, The Catholic University of Korea, Incheon 21431, Korea; leoanzel@catholic.ac.kr

**Keywords:** renin, angiotensin II, normal tension glaucoma, renin–angiotensin–aldosterone system

## Abstract

The purpose of this study was to investigate the function of the renin–angiotensin–aldosterone system (RAAS) in normal tension glaucoma (NTG) patients by measuring the level of renin and angiotensin II (AngII) in the plasma. Twenty-four patients with NTG and 38 control subjects were included in this study. Renin and AngII were measured in the blood samples of all subjects by enzyme-linked immunosorbent assay (ELISA). No significant differences were found in the complete blood count, fasting glucose, low-density lipoprotein (LDL), and high-sensitivity C-reactive protein (hs-CRP) levels between the control and NTG groups. The systemic concentration and variability of the renin concentration in the blood was significantly higher in the NTG group (*p* = 0.005 and 0.005, respectively). According to multivariate logistic regression analysis, the variability of the renin concentration was associated with NTG (*p* = 0.006). In conclusion, the systemic concentration and variability of renin levels were elevated in NTG patients. An altered renin concentration could represent a difference in RAAS function in NTG patients.

## 1. Introduction

Glaucoma is characterized by the progressive death of retinal ganglion cells (RGCs) and the associated visual field (VF) defect. Elevated intraocular pressure (IOP) is traditionally known to be the main cause of glaucoma [1]. However, some glaucoma patients with normal IOP have shown similar clinical features to patients with elevated IOP. In Asia, glaucoma patients with normal IOP are the predominant type [2,3].

Glaucoma patients with normal IOP, known as normal tension glaucoma (NTG), have distinct clinical features associated with vascular dysregulation. According to several studies, orthostatic hypotension, systemic arterial hypotension, Raynaud’s phenomenon, and migraines are common in NTG patients [4,5,6]. According to a population-based study, fluctuation of systolic blood pressure (BP) is a contributing factor to the development of open angle glaucoma (OAG) [7]. Considering that this study was conducted in Korea, where most OAG patients have normal IOP, this result also supports the association of systemic hemodynamic instability with NTG.

The renin–angiotensin–aldosterone system (RAAS) is important for regulating hemodynamic stability and fluid volume in the body [8]. When hypotension occurs with dysregulated vascular status, the RAAS is activated and renin is released from the kidneys. The released renin acts on angiotensinogen to generate angiotensin II (AngII), which is the physiologically active form [9]. AngII, which is mediated via AngII receptors, has multiple effects, including helping to maintain a constant fluid volume in the body [10,11]. Therefore, we hypothesized that NTG patients with a high probability of hemodynamic instability could have an altered RAAS function compared to normal controls.

The purpose of this study was to investigate the systemic hemodynamic features associated with RAAS function in patients with NTG. To the best of our knowledge, no study has been conducted measuring renin and AngII levels in the blood of NTG patients. We measured the concentration of renin and AngII, as well as the complete blood count, fasting glucose, total cholesterol, high-density lipoprotein (HDL), and low-density lipoprotein (LDL) levels in the blood of the study subjects. The results of the plasma concentrations of renin and AngII, including the variability, were compared to those of normal controls.

## 2. Materials and Methods

### 2.1. Study Design and Population

This study was performed according to the tenets of the Declaration of Helsinki and was approved by the Institutional Review and Ethics Board of Incheon St. Mary’s Hospital, South Korea. All subjects of this single-center, case–control study provided written, informed consent. Normal controls were recruited from a routine ocular examination group, and patients with NTG were recruited from the glaucoma clinic of Incheon St. Mary’s Hospital. Patients were excluded if they met any of the following criteria: (1) a history of non-glaucomatous optic neuropathy; (2) a history of eye trauma or surgery, except for uncomplicated cataract extraction; (3) pathologic myopia (i.e., chorioretinal atrophy, intrachoroidal cavitation, choroidal neovascularization, and lacquer crack); (4) other retinal diseases, including diabetic retinopathy and retinal vascular diseases such as vascular occlusion or uveitis.

All subjects underwent comprehensive ophthalmic examinations, including best-corrected visual acuity (BCVA), refraction, Goldmann applanation tonometry, slit-lamp examination, gonioscopy, and dilated fundus bimicroscopy. Fundus photography, stereoscopic photography of the optic disc (Kowa, VK-2, Torrance, CA, USA), and perimetry with the Swedish Interactive Threshold Algorithm (SITA) 24-2 test on a Humphrey Field Analyzer were performed to diagnose NTG. The diagnostic criteria for NTG were as follows: (1) open angle by gonioscopy; (2) IOP <22 mmHg; (3) glaucomatous-appearing optic disc morphology corresponding to glaucomatous VF defects on SITA 24-2 results, as follows: a glaucomatous-appearing optic disc accompanied by increased cup-disc ratio (CDR) of more than 0.7, a difference in vertical CDR of more than 0.2 between both eyes, retinal nerve fiber layer (RNFL) defects, or neural rim thinning. We defined glaucomatous VF defects as meeting two of the following three criteria: (1) a cluster of 3 points with a probability lower than 5% on the pattern deviation map in at least one hemifield and including at least 1 point with a probability lower than 1% or a cluster of 2 points with a probability lower than 1%; (2) glaucoma hemifield test results outside normal limits; (3) a pattern standard deviation outside 95% of the normal limits. VF defects were confirmed by at least two consecutive and reliable tests (defined as a false-negative rate of <15%, a false-positive rate of <15%, and fixation losses of <20%). All NTG patients had anti-glaucomatic eyedrops and showed stable disease status without VF progression for three years.

BP was measured after the study subjects had rested in a sitting position for 5 min. Trained clinicians took measurements of two consecutive systolic and diastolic BPs, and the average value was used. For hypertension patients, the type of hypertensive medication was identified, and subjects with angiotensin-converting enzyme (ACE) inhibitors or angiotensin II receptor blockers (ARBs) were excluded, as these medications could interfere with systemic renin and angiotensin II levels.

### 2.2. Measurement of Renin and Angiotensin II with Enzyme-Linked Immunosorbent Assay (ELISA)

After fasting for 12 h and resting for 30 min, blood samples for the biochemical analysis were obtained by standard venipuncture. Blood was collected into tubes containing EDTA as an anticoagulant for plasma preparation. As soon as a blood sample was collected, the tube was frozen at −20 °C, and repeated freeze–thaw cycles were avoided. Frozen samples were thawed before conducting ELISA of renin (R&D Systems, Minneapolis, MN, USA) and EIA of AngII (Phoenix Pharmaceuticals, Inc., Burlingame, CA, USA) using commercially available kits and according to the manufacturer’s instructions. In brief, standards, samples, and controls were added to each well of a microplate pre-coated with a secondary antibody specific for renin and AngII. After incubation and washing the plate, a primary antibody was added, and the plate was again incubated and washed. Subsequently, a chromogenic substrate was added to each well for the development of color, and the intensity was measured by a microplate reader. All measurements were assayed in duplicate and the mean values were calculated. In addition, the standard deviations (SDs) of the measurements were calculated to assess the variability of renin and AngII.

### 2.3. Statistical Analysis

All data are expressed as mean ± standard deviation. Independent *t*-tests were used to compare the continuous variables between the control and NTG groups. Chi-square tests were used to compare the categorical variables between the two groups. To identify the factors associated with NTG, logistic regression analyses were performed. Variables with *p*-values <0.1 in the univariate analyses were included in the multivariate analysis. The odds ratio (OR) and 95% confidence interval (CI) were calculated for each variable. All statistical analyses were performed with SPSS version 24.0 (IBM Corp., Armonk, NY, USA). *p* < 0.05 was considered statistically significant.

## 3. Results

This study included 24 patients with NTG and 38 controls as study subjects. Table 1 shows the clinical characteristics of the subjects. There were no significant differences in age, gender, the ratio of hypertension and diabetes, or blood pressure between the two groups. Ocular characteristics such as BCVA, spherical equivalent, and IOP did not differ significantly between the two groups.

The laboratory findings were comparable between the two groups, as shown in Table 2. White blood cell (WBC) count, red blood cell (RBC) count, hemoglobin, hematocrit, platelet count, fasting glucose, LDL, and hs-CRP were not significantly different between the control and NTG groups. With respect to the lipid profile, total cholesterol was higher (*p* = 0.027) and HDL was lower (*p* = 0.036) in the NTG patients.

**Figure 1:**
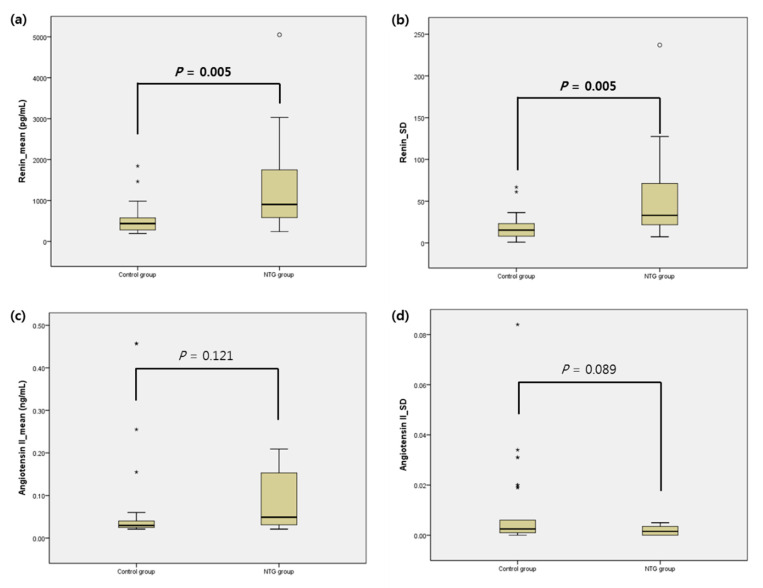
Graphs representing the mean value and standard deviation (SD) of renin and angiotensin II from the plasma of the control and NTG groups. The midline of the box plot represents the mean, while the bar of each box shows the 95% confidential interval. Dots and stars identify the outliers. Outliers of the mean and SD of angiotensin II in the NTG group could not be marked in the graph due to a limitation of scale. In angiotensin II, the mean values of the outliers were 1.84, 3.82, and 10.5. In angiotensin II, the SD values of outliers were 0.516, 0.322, 0.328, and 0.225. Statistical significance was compared to the control group and significant values are shown in bold. (**a**) Renin_mean (pg/mL), (**b**) Renin_SD, (**c**) Angiotensin II_mean (ng/mL), (**d**) Angiotensin II_SD.

The mean concentration and SD of renin and AngII from each group are reported in Table 3 and Figure 1. The mean concentration and SD of renin were higher in the NTG group (mean: 528.06 ± 341.54 vs. 1355.64 ± 1279.52, *p* = 0.005; SD: 17.79 ± 14.29 vs. 49.45 ± 49.41, *p* = 0.005). The mean concentration and SD of AngII were higher in the NTG group but not statistically significant (*p* = 0.121 and 0.089, respectively). According to Figure 1, the range of distribution of renin was wider in the NTG group.

The factors related to NTG from the clinical and laboratory findings were evaluated using logistic regression analyses (Table 4). In the univariate analyses, the total cholesterol, mean value, and SD of renin were associated with NTG (*p* = 0.033, 0.003, and 0.002, respectively). In the multivariate analysis, including the variables with *p*-values ≤0.10 from the univariate analyses, only the SD of renin was significantly associated with NTG (*p* = 0.006).

## 4. Discussion

The systemic features representing hemodynamic instability include systemic arterial hypotension, orthostatic hypotension, and a nocturnal BP dip. These features have been reported as commonly found characteristics in NTG patients [12,13,14]. A population-based study reported that lower systolic BP doubles the risk of glaucoma development, independent of the effect of IOP [15]. According to the study of Choi et al., a nocturnal BP dip is associated with circadian ocular perfusion pressure disturbance, and disturbed ocular perfusion could affect the development of NTG [13]. Park et al. suggested that orthostatic hypotension is a significant factor associated with NTG progression [16].

When systemic hypotension occurs in the body, the RAAS starts to regulate the blood volume and systemic vascular resistance [17]. The RAAS functions to maintain the blood pressure through elevating the arterial tone and blood volume. In response to lowered BP, activated juxtaglomerular cells of the kidney cleave prorenin to renin. Increased renin causes the sequential cleavage of angiotensinogen to angiotensin I and II [18].

In the present study, we found that the systemic mean concentration and variability of renin was higher in the NTG patients. Additionally, the SD of the renin concentration was a meaningful factor associated with NTG. AngII was thought to be elevated in response to an increase in renin; however, there was no statistically significant difference between the NTG and control groups. In short, this study identified a link between systemic RAAS functioning and NTG through the measurement of the systemic concentration of renin and AngII. RAAS function showed a different pattern in the NTG patients when compared to normal controls.

According to our previous study, the systemic concentration of endothelin-1 (ET-1) and macrophage chemoattractant protein-1 (MCP-1) are elevated in NTG patients [19]. ET-1 is widely known as a mediator of inflammation, contributing to vascular dysfunction [20]. The chemotactic activity of MCP-1 can also cause diverse vasculopathy by diapedesis of monocytes [21]. These factors associated with vascular dysfunction might explain the disturbed fluid volume and RAAS function, leading to the rise of renin and angiotensin II levels, to some extent.

In healthy eyes, retinal blood flow is mediated by endothelial cells because there is no autonomic nervous system innervation [22]. AngII, affected by the release of renin as a constituent of the RAAS, is one of the mediators controlling the contraction and relaxation of vascular endothelium [23]. A previous study using animal models reported that angiotensin plays a role in the regulation of ophthalmic microcirculation [24]. A weakened blood–brain barrier of the optic nerve head in glaucoma [25] could pass systemic RAAS components and may have an influence on the retinal microcirculation.

In addition, most RAAS components and receptors were found in the retinal tissue. The local RAAS from tissue, including the retina, has been reported as a system that maintains the homeostasis of tissue [26]. Prorenin receptors that could result in renin having enzymatic activity were reported as important molecules in the RAAS of tissue. Activated renin and the resultant angiotensin elevation could function as a stimulator of inflammatory cascades in the neural tissue of the retina [27]. There have been several studies reporting the role of neuroinflammation in glaucoma [28,29,30], and the RAAS could be a link between vascular dysregulation and neuroinflammation-associated RGC death in glaucoma.

In this study, total cholesterol and HDL were different between the two groups. The NTG group had worse lipid profiles in terms of dyslipidemia. However, lipid profiles were not independently associated with NTG in the regression analyses. Indeed, there is controversy regarding the relationship between dyslipidemia and glaucoma. Wang et al. suggested that hyperlipidemia is related to an increased risk of glaucoma and IOP elevation [31]. It has also been reported that higher serum cholesterol is related to a higher risk of glaucoma [32]. However, according to a population-based study in Korea, there is no significant association of total cholesterol and HDL with glaucoma [33]. Our study is limited due to the small number of subjects; thus, future studies should be conducted with larger numbers of subjects and various populations.

The other limitations of this study should be mentioned. First, clinical features associated with vascular dysregulation, such as optic disc hemorrhage, migraine, and Raynaud’s phenomenon, were not included in our analysis. Since normal controls were recruited from routine medical examinations, information on their hemodynamic features was not available. Several studies have already reported the correlation of vascular dysregulation with optic disc hemorrhage [34], migraines [35], and Raynaud’s phenomenon [36]. For further understanding, it is necessary to study these comprising factors and to investigate their effect on RAAS function in NTG patients. Second, since we included only subjects with NTG, it is hard to generalize these results to all types of glaucoma. High-pressure glaucoma has different hemodynamic systemic characteristics to NTG [37]; therefore, it is unreasonable to extend the distinct RAAS status to patients with other types of glaucoma. Furthermore, because all subjects had normal IOP, it was hard to determine the effect of IOP on RAAS function. For further understanding, studies comprising glaucoma patients with elevated IOP should be performed with a larger number of subjects. Finally, the short half-life degradation time of AngII could have prevented the accurate measurement of the plasma. Though freezing was performed immediately after the blood sample was taken to minimize measurement errors, the sample could still have been affected by the half-life time. This could be a compromising factor that resulted in the nonsignificant results for AngII in contrast to renin.

In conclusion, NTG patients showed elevation and fluctuation of systemic renin concentration. The difference in RAAS function in NTG patients may be associated with diverse features of vascular dysregulation. Further studies investigating the relationship between RAAS and NTG could suggest a novel approach for disease modulation.

## Figures and Tables

**Table 1:** Comparison of the clinical characteristics of the control group and the normal tension glaucoma (NTG) group (*n* = 62).

Clinical Characteristics	Control (*n* = 38)	NTG (*n* = 24)	*p*-Value
Age (years)	67.97 (±10.85)	67.61 (±15.58)	0.920
Sex (male/female)	15/23	14/10	0.147
Hypertension (%)	12 (31.6)	11 (45.8)	0.258
Diabetes (%)	8 (21.1)	2 (8.3)	0.185
BCVA (decimal)	0.77 (±0.25)	0.71 (±0.27)	0.297
Spherical equivalent (diopter)	−1.05 (±1.56)	−1.95 (±2.64)	0.162
Intraocular pressure (mmHg)	12.94 (±2.89)	12.41 (±3.61)	0.526
Systolic blood pressure (mmHg)	131.28 (±18.72)	128.17 (±9.17)	0.425
Diastolic blood pressure (mmHg)	75.17 (±9.39)	76.58 (±7.93)	0.595

BCVA: best corrected visual acuity.

**Table 2:** Comparison of the blood examination of the control group and the normal tension glaucoma (NTG) group.

Examination Results	Control (*n* = 38)	NTG (*n* = 24)	*p*-Value
WBC (10^9^/L)	6.37 (±2.54)	7.33 (±3.01)	0.184
RBC (10^12^/L)	4.36 (±0.47)	4.26 (±0.60)	0.474
Hemoglobin (g/dL)	13.44 (±1.28)	13.13 (±1.71)	0.429
Hematocrit (%)	40.41 (±3.45)	39.71 (±5.07)	0.557
Platelet (10^9^/L)	256.28 (±83.46)	257.62 (±69.89)	0.948
Fasting glucose (mg/dL)	116.25 (±28.66)	104.39 (±16.20)	0.070
Total cholesterol (mg/dL)	170.78 (±38.69)	199.41 (±49.81)	**0.027**
HDL (mg/dL)	52.14 (±7.53)	42.50 (±1.73)	**0.036**
LDL (mg/dL)	108.53 (±34.21)	112.00 (±34.49)	0.729
Hs-CRP (mg/dL)	1.62 (±2.83)	3.58 (±5.52)	0.092

WBC: white blood cell; RBC: red blood cell; HDL: high-density lipoprotein; LDL: low-density lipoprotein; hs-CRP: high-sensitivity C-reactive protein. Significant values are shown in bold.

**Table 3:** Results of the enzyme-linked immunosorbent assay (ELISA) of renin and angiotensin II in the control group and the normal tension glaucoma (NTG) group.

ELISA results	Control (*n* = 38)	NTG (*n* = 24)	*p*-Value
Renin_mean (pg/mL)	528.06 (±341.54)	1355.64 (±1279.52)	**0.005**
Renin_SD	17.79 (±14.29)	49.45 (±49.41)	**0.005**
Angiotensin II_mean (ng/mL)	0.14 (±0.38)	0.91 (±2.31)	0.121
Angiotensin II_SD	0.01 (±0.01)	0.05 (±0.13)	0.089

SD: standard deviation. Significant values are shown in bold.

**Table 4:** Systemic factors associated with normal tension glaucoma (NTG).

Variables	Univariate			Multivariate		
	Exp(ß)	95% CI	*p*-Value	Exp(ß)	95% CI	*p*-Value
Age (years)	0.998	0.953 to 1.044	0.918			
Female gender (vs. male)	0.466	0.165 to 1.318	0.466			
Hypertension	1.833	0.638 to 5.264	0.260			
Diabetes	0.341	0.066 to 1.765	0.200			
Systolic BP	0.987	0.950 to 1.026	0.515			
Diastolic BP	1.019	0.953 to 1.089	0.587			
WBC	1.138	0.935 to 1.383	0.197			
RBC	0.693	0.258 to 1.864	0.468			
Platelet	1.000	0.994 to 1.007	0.947			
Total cholesterol	1.015	1.001 to 1.029	**0.033**	1.017	1.000 to 1.034	0.052
Fasting glucose	0.977	0.951 to 1.004	0.101			
HDL	0.634	0.311 to 1.295	0.211			
LDL	1.003	0.986 to 1.020	0.723			
Hs-CRP	1.126	0.971 to 1.305	0.117			
Renin_mean	1.002	1.001 to 1.003	**0.003**			
Renin_SD	1.058	1.021 to 1.096	**0.002**	1.051	1.015 to 1.088	**0.006**
Angiotensin II_mean	1.951	0.838 to 4.544	0.121			
Angiotensin II_SD	1.093	0.980 to 1.219	0.110			

BP: blood pressure; WBC: white blood cell; RBC: red blood cell; HDL: high-density lipoprotein; LDL: low-density lipoprotein; hs-CRP: high-sensitivity C-reactive protein; SD: standard deviation. Significant values are shown in bold.
